# Pilot Study of 15 Patients Receiving a New Treatment Regimen for Androgenic Alopecia: The Effects of Atopy on AGA

**DOI:** 10.5402/2011/241953

**Published:** 2011-04-11

**Authors:** A. W. Rafi, R. M. Katz

**Affiliations:** HAIR CYNR-G, INC., 11500 West Olympic Boulevard, No. 630, Los Angeles, CA 90064, USA

## Abstract

*Background*. We examined the efficacy of a new regimen to treat AGA, with attention to male patients who are atopic. *Objective*. To assess the efficacy of a four-part regimen for the treatment of AGA in atopic and nonatopic patients. NuH Hair is a novel topical combination of finasteride, dutasteride, and minoxidil, which is blended in a hypoallergenic lotion. The other three components included Rogaine foam, Propecia, and ketoconazole shampoo. *Methods*. A prospective pilot study was conducted in 15 patients. All patients were assessed for the presence of atopy. Each patient served as their own control. All patients were treated specifically with NuH Hair and were given the option to add any of the other components of the protocol to their regimen. Photographs were taken of each patient's scalp at months 0, 1, 3, 6, and 9. *Results*. All 15 patients demonstrated significant growth of hair. In those patients who utilized all 4 components, significant growth was achieved in as little as 30 days. In those patients who choose only to utilize NuH Hair, significant growth was demonstrated after 3 months. *Conclusion*. Aggressively treating AGA achieves significant and rapid growth of new hair. This is effective in atopic and nonatopic male patients.

## 1. Introduction

Male pattern baldness (MPB), also called androgenetic alopecia (AGA), is the most common form of alopecia found in men. It affects approximately 30% of men by the age of 30, 50% of men by age 50, and 57% of men by age 60 [1–4]. MPB is in part genetically determined and is potentially reversible. With MPB, one experiences miniaturization of the hair follicles and shortening of the anagen (growth) phase in the involved hairs. The main androgen responsible for these changes to the hair follicle is dihydrotestosterone (DHT). Testosterone is converted to DHT by an enzyme, 5-alpha reductase (5AR). Men who have a congenital deficiency of 5AR (type II) do not experience MPB [5–9]. 

Most treatment modalities for MPB are not FDA approved and overall not significantly effective. The high prevalence of MPB, significant early age of onset, and large degree of associated psychosocial morbidity have created a large market for MPB treatment. Despite the paramount demand for MPB treatment, there are only two FDA-approved medications. However, they are costly, require lifelong treatment, and may have side effects.

The two FDA-approved medications used to treat MPB include topical minoxidil (Rogaine c; Johnson and Johnson) and finasteride (Propecia c; Merck). Minoxidil is a vasodilator which is directly applied to the scalp to stimulate growth of the hair follicles. Topical minoxidil slows hair loss for many men, while in some men, it grows new hair. The previous degree of hair loss returns when solution application is discontinued. Minoxidil is available in 2% and 5% solutions, and a 5% foam version which became available in 2007. In addition, Propecia 1 mg (oral) was approved by the FDA in 1998 for AGA. Propecia (finasteride) is a prescription pill that inhibits the production of the male hormone dihydrotestosterone (DHT). This is done by primarily blocking 5AR (type 2), the enzyme, which converts testosterone to DHT. Like minoxidil, it is likely to slow hair loss but can also stimulate new hair growth. In general, it is somewhat more effective than minoxidil, and Propecia c is even more effective when combined with minoxidil. As with minoxidil, one's previous degree of hair loss returns when Propecia c is discontinued.

There are other medications available on the market, which claim to treat AGA. These medications are, however, not FDA approved for MPB. Ketoconazole, (Nizoral) shampoo, is also known to inhibit 5AR, the enzyme which converts testosterone to DHT. Preliminary research suggests that ketoconazole shampoo may be beneficial in men suffering from androgenic alopecia [10, 11]. Support for this also stems from a study in 1998 which compared ketoconazole shampoo 2% to the proven hair loss drug minoxidil in men with androgenic alopecia [10]. The study concluded that hair density, size, and proportion of anagen follicles were improved almost similarly by both ketoconazole and 2% minoxidil regimens. In addition, since ketoconazole effectively treats the Pityriasis (also called Malassezia) fungus that commonly inhabits the scalp, it was hypothesized that it may prevent hair loss by reducing inflammation from the fungus, in addition to having antiandrogenic properties. The researchers were guarded about the meaning of their results. They suggested that more rigorous studies on larger groups of men should be done to confirm their findings and to evaluate the ideal dosage, formulation, and desirability of routine treatment. Although no further research in humans has been undertaken, a study on ketoconazole in 2005 corroborated the existence of a stimulatory effect on hair growth in mice [12]. 

In patients who are atopic, there is an increased susceptibility to infection or colonization with various organisms. For example, *Staphylococcal aureus* is detected in more than 90% of skin lesions of atopic dermatitis (AD). A superimposed dermatophytosis including *Trichophyton rubrum* and *Malassezia furfur (Pityrosporum ovale)* can cause flares of atopic dermatitis. The rate of isolation of Malassezia from the skin of AD patients is much higher than from the skin of healthy control patients without atopy. This fungal (yeast) organism is also commonly associated with concomitant seborrheic dermatitis in atopic patients. *Malassezia furfur* is a lipophilic yeast, and antiyeast IgE antibodies have been found in patients with a predominant head and neck eczematous dermatitis. This common presentation in atopic dermatitis patients provided the interest in studying our patients with clinical fungal infection of the head and neck. Thus, it provides significant anecdotal evidence for the correlation of a superimposed fungal infection of the scalp in patients with seborrheic dermatitis and MPB [13].

Anecdotal reports indicate that both the 1% and 2% dosages of ketoconazole shampoo have hair loss benefits; however, the more potent 2% formulation likely produces better results. Excessive usage of either formulation has not been shown to produce better results. The results produced in this study were based on ketoconazole 2% shampoo, used once every 2–4 days, and leaving the shampoo on the scalp for 3–5 minutes before rinsing (as is recommended with the treatment of dandruff and seborrheic dermatitis). It has been stated that medications capable of maintaining the existing hair population, even in the absence of hair regrowth, should be regarded as effective treatments for androgenic alopecia. Ketoconazole shampoo is only FDA approved for the treatment of dandruff and seborrheic dermatitis of the scalp. Therefore, it cannot be endorsed or marketed as a hair loss remedy to the general public [11].

Another important medication which inhibits 5AR is dutasteride, (Avodart-GSK). There has been significant attention towards dutasteride as it is the only known medication which blocks both types of 5-alpha reductase (types I and II). Type I 5AR is the principle isoenzyme found in sebaceous and sweat glands and the scalp [5, 14–19], whereas type II 5AR is present in hair follicles and the prostate [14]. In addition to blocking both isoenzymes of 5AR (I and II), dutasteride has been shown to be 3 times more potent then finasteride at inhibiting type II 5AR and more than 100 times potent at inhibiting type I isoenzyme. Oral dutasteride also decreases serum DHT by upto 90%, whereas finasteride only reduces concentrations of serum DHT by 70% [5, 20]. These properties make dutasteride a more ideal candidate for treating MPB. Despite these more desirable properties, limited research using dutasteride for MPB treatment has been undertaken. 

Initially, phase I and II clinical trials for dutasteride as a hair loss drug were undertaken, but called off in late 2002. The reason these trials were called off is not publicly known. Industry sources speculate that dutasteride would have been seen as too similar to Propecia (1 mg/day finasteride) if have been proven profitable on the market as a hair loss treatment [5]. However, phase II study results indicated that oral dutasteride at both 0.5 mg and 2.5 mg/day generated a superior hair count to finasteride 5 mg at 12 and 24 weeks (Olsen et al.) [5]. In this study, 3 different doses of dutasteride were equal to or more effective than finasteride at increasing hair counts. The most effective dose was found to be 2.5 mg of dutasteride daily, however, more side effects were seen in this group. Quantitative hair counts were measured at 24 weeks into the study in all patients. Hair counts in the placebo group was −32.3 hairs, in the finasteride 5 mg/day group was +75.6 hairs, in the dutasteride 0.1mg/day group was +78.5 hairs, in the dutasteride 0.5 mg/day group was +94.6 hairs, and in the dutasteride 2.5 mg/day group was +109.6 hairs [5].

## 2. Methods

**Figure 1:**
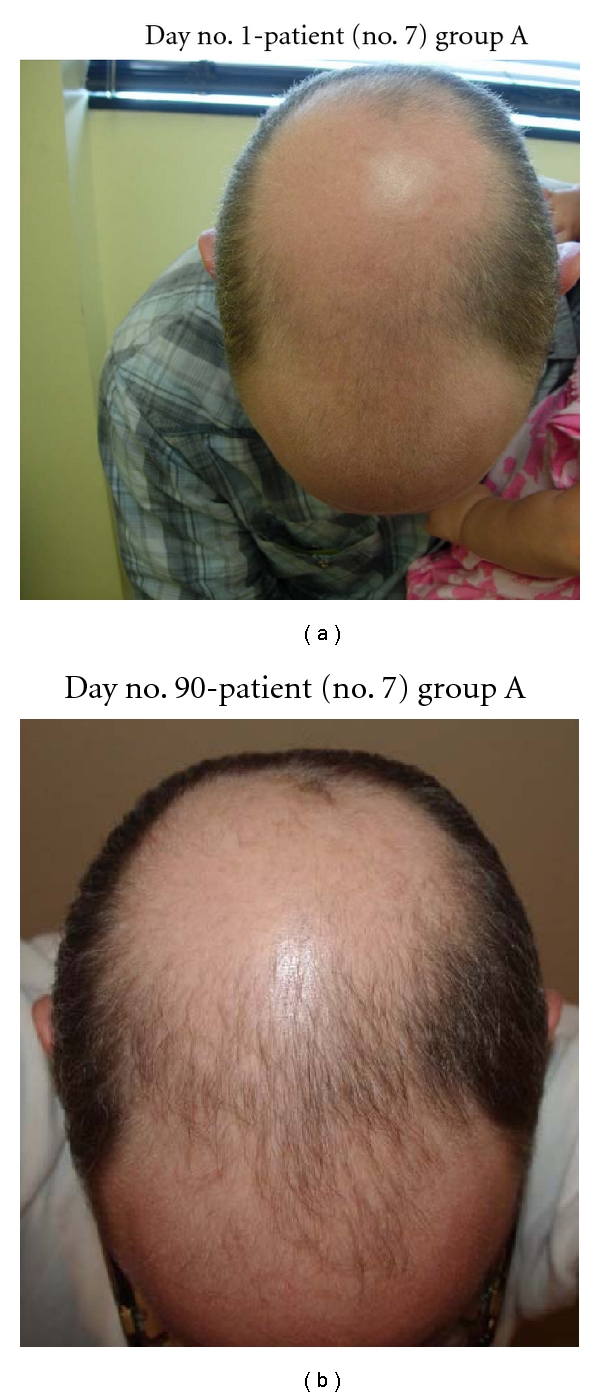
(a) Note patient no. 7 with stage 7 AGA on Hamilton Norwood Scale. (b) Note substantial growth at frontal scalp with new thick and more dense hairs.

15 male patients from our Allergy and Immunology practice with AGA were followed over a 9-month period, from April 2010 to December 2010. Patients' ages ranged from 24 years to 72 years old (Table 1). This study included 10 atopic patients and 5 non-atopic patients, and each patient was assessed for evidence of seborrheic dermatitis. Patients with varying degrees of hair loss were assessed based upon the Hamilton Norwood Scale (Figures 1 and 17), and all patients were recommended to implement aggressive treatment for seborrheic dermatitis.

Our research product (NuH Hair) formulation is currently a trade secret. The overall contents, composition percentages, and formulation methods are not able to be revealed.

All patients were started on NuH Hair applied one time per day. All patients were given the option to add the three other components to our hair regrowth protocol which included the following.

- Propecia (Merck) 1 mg taken daily. Propecia was included in our protocol because it is currently the only oral 5-alpha reductase inhibitor on the market which is FDA approved.
- Rogaine (Johnson & Johnson Healthcare Products — Division of McNeil) Foam 5% applied at least once per day.
- Nizoral (ketoconazole) 2% shampoo applied 2-3 times per week. This shampoo came with strict instructions for patients to scrub into the scalp for 100 seconds and then let the foam remain on the scalp for 10 minutes prior to washing out, for each application.

All patients had documentation via photographs taken of their scalp prior to starting their medication regimen. Each patient served as their own control. All patients had additional photographs taken of their scalp at months 1, 3, 6, and 9. All patients were assessed to follow medication compliance, and each patient was seen on a monthly basis.

## 3. Results

**Figure 2:**
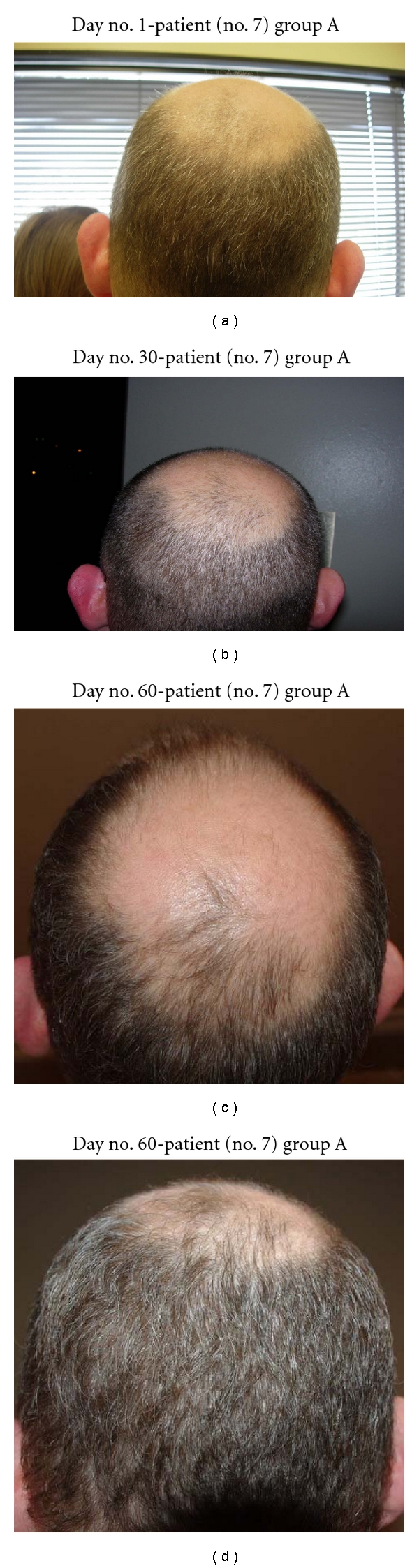
(a) Note that the vertex of scalp is nearly void of hair. (b) At day no. 30, though hair is overall shorter, there are new hairs growing in the vertex. (c) At day no. 60, many new hairs were found at the vertex and the frontal scalp. The overall area on top of the head which was originally void of hair has decreased in size. (d) Another view of the vertex demonstrates substantial hair growth.

**Figure 3:**
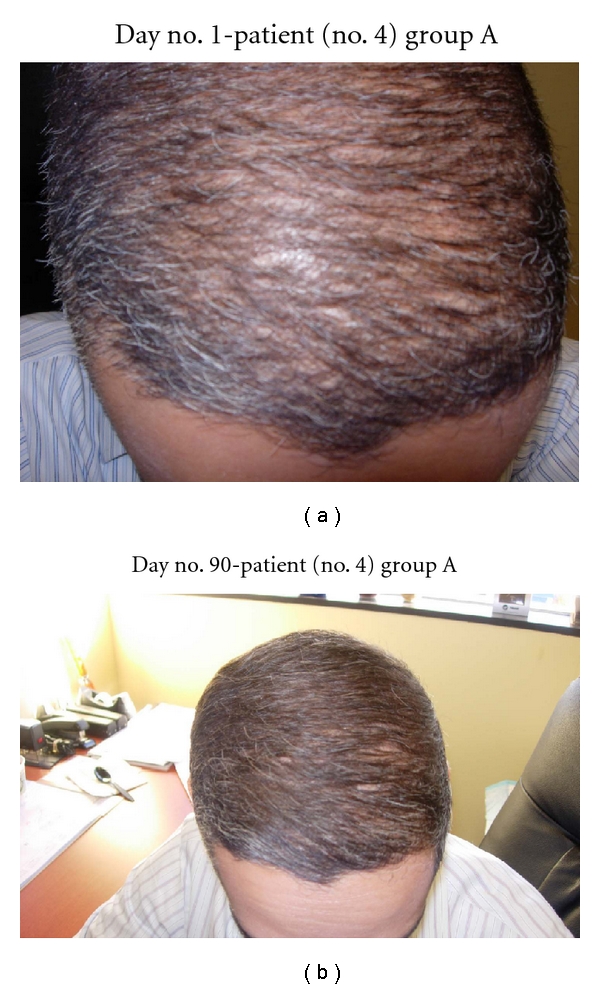
(a) Frontal scalp demonstrates overall thin hair distribution. (b) At day no. 90, this patient's hair density has increased significantly and diffusely.

**Figure 4:**
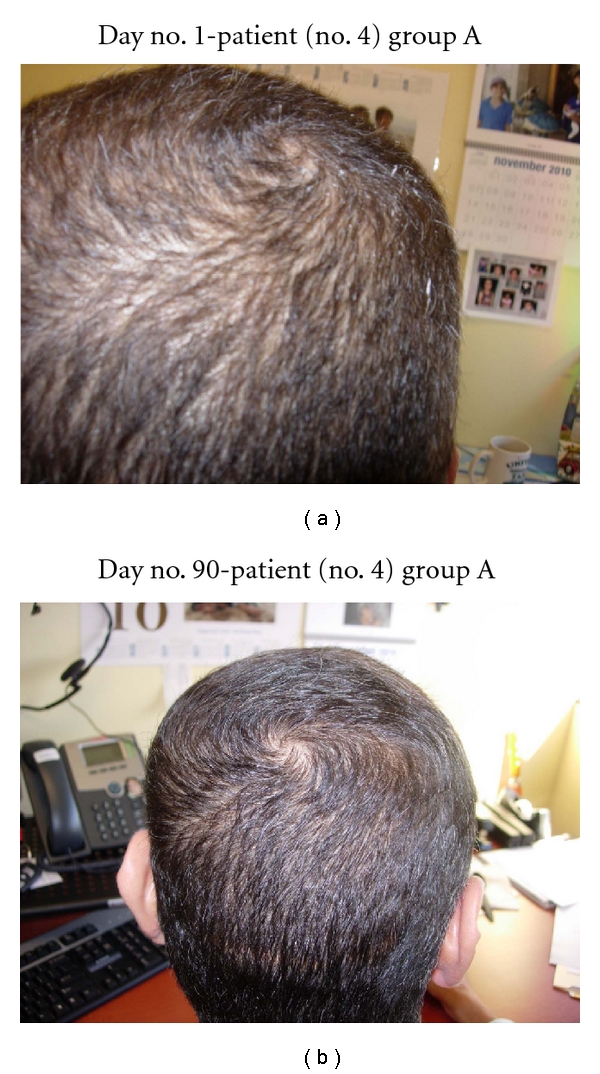
(a) Vertex demonstrates minimal thinning of hair overall. (b) At day no. 90, the vertex demonstrates very thick hair growth.

**Figure 5:**
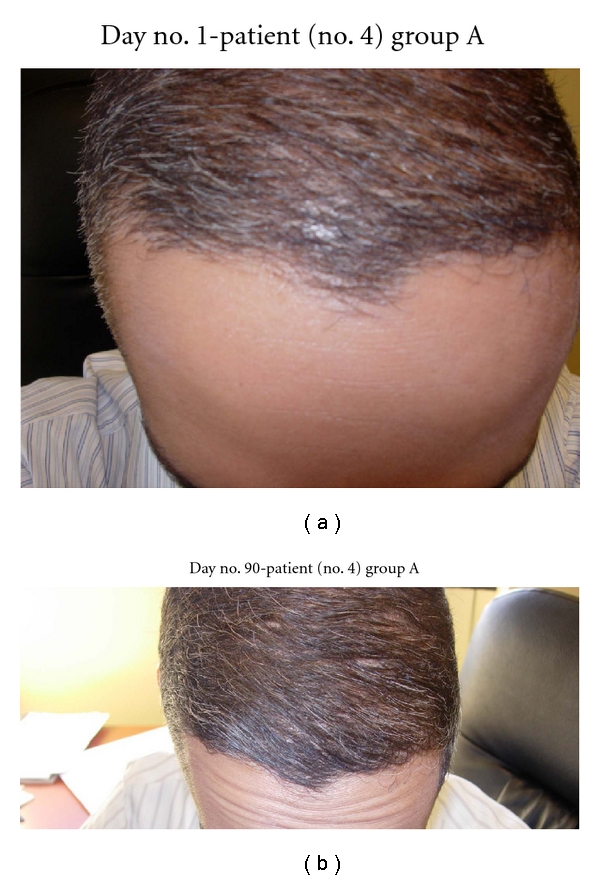
(a) Patient no. 4's hairline has thinning areas. (b) At day no. 90, this patient's hairline is much more defined and thicker.

**Figure 6:**
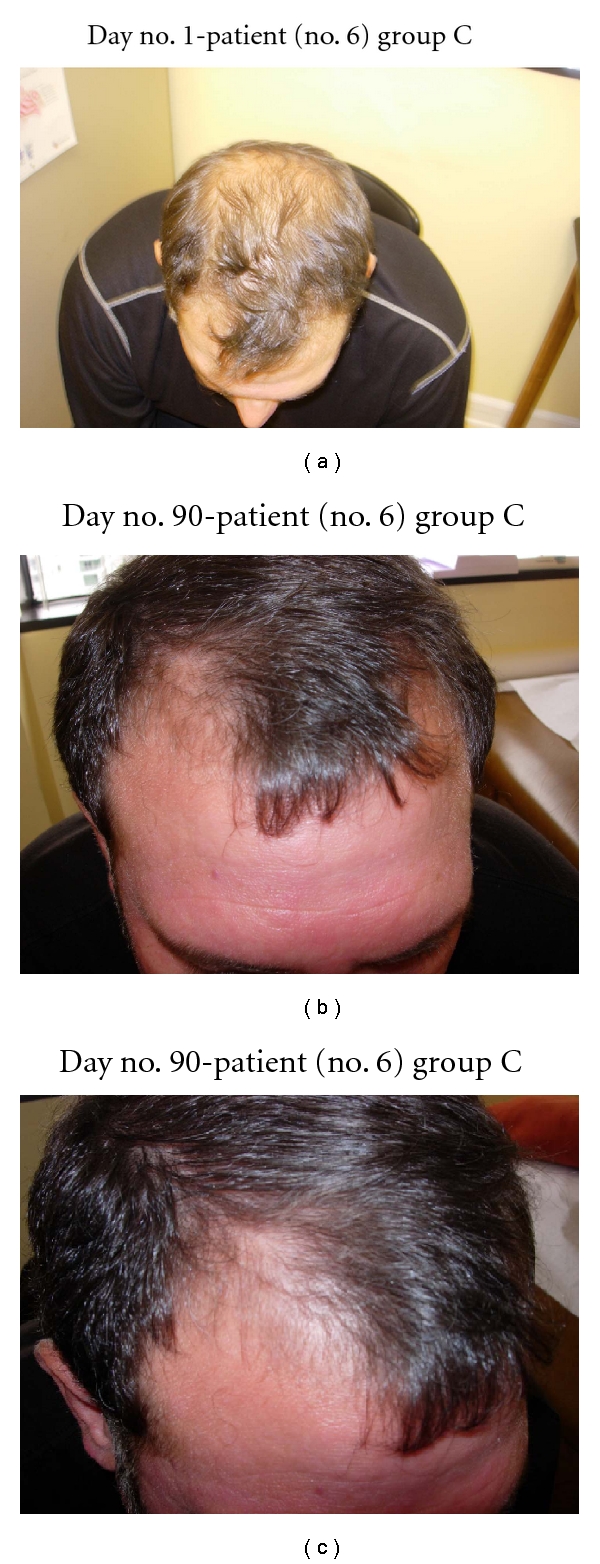
(a) Diffuse thinning of the frontal scalp and vertex is noted. (b) At day no. 90, thick hair growth is noted on the frontal scalp. (c) The frontal scalp and temporal scalp hairs are becoming dense.

**Figure 7:**
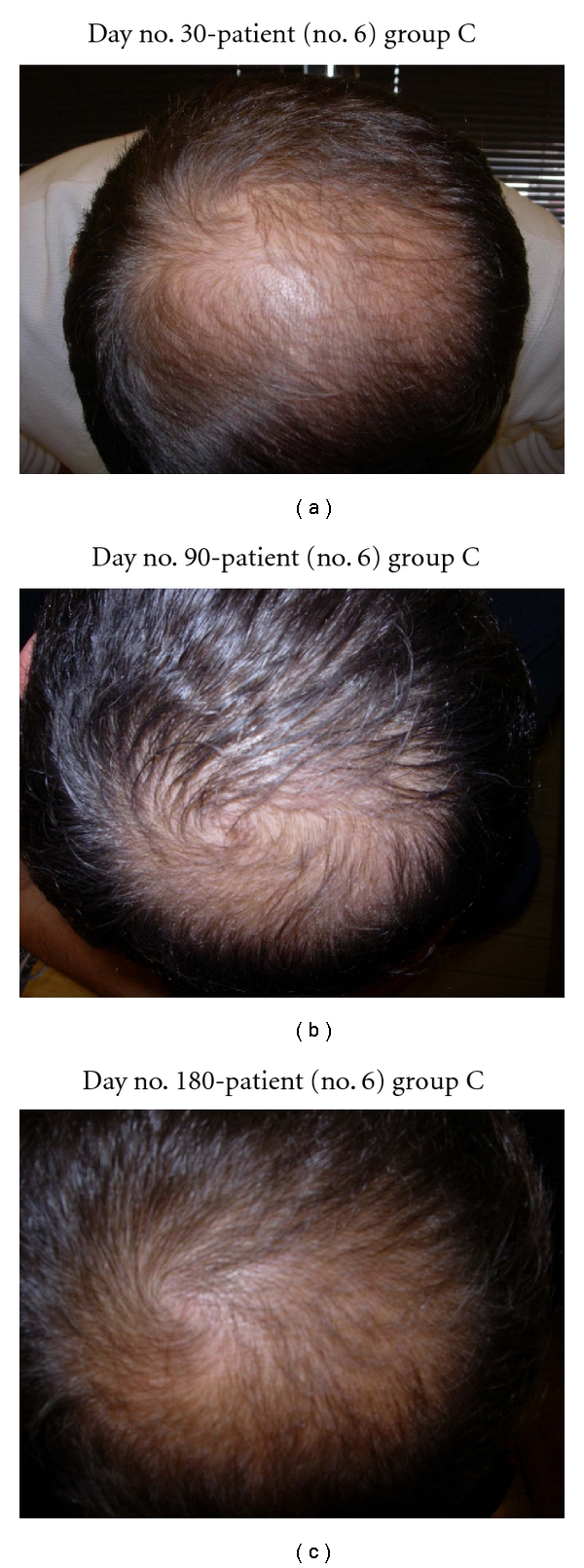
(a) Scalp vertex at day no. 30 already shows growth. (b) Scalp vertex at day no. 90. (c) Scalp vertex at day no. 180 (6 months) demonstrates major growth.

**Figure 8:**
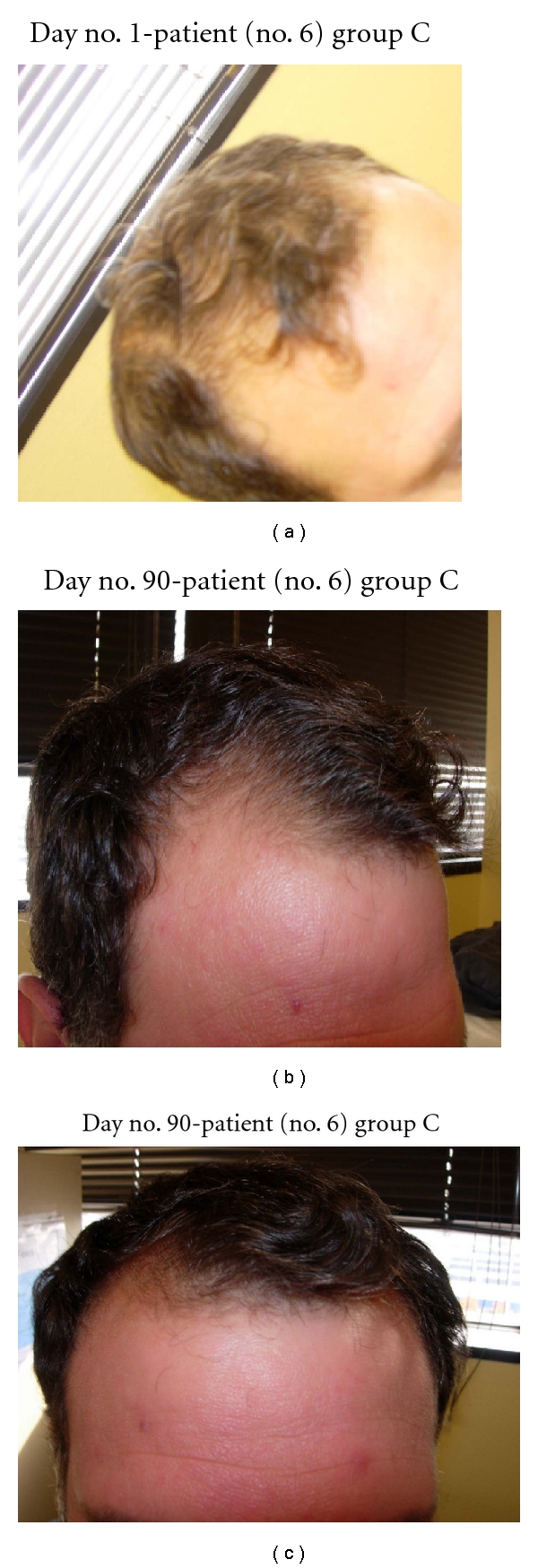
(a) Anterior scalp demonstrates major thinning and poorly defined hairline. (b) Frontal scalp and hairline are growing in at day no. 90. (c) Frontal scalp and hairline are much thicker at day no. 90.

In this study, all 15 patients demonstrated significant hair regrowth using this research protocol. In addition, all patients reported subjective improvement of the health of their scalp, and this was confirmed on their followup physical examinations. All patients also noted that their pre-existing hair grew much quicker than it did prior to starting their protocol. All patients who demonstrated signs of seborrheic dermatitis (SD) reported significant improvement of their SD. This was also confirmed at their followup examination. Eight (8) patients (group A) used all 4 components of the research protocol, five (5) patients used only NuH Hair (group D), one (1) patient (group C) used NuH Hair and 2% ketoconazole shampoo, and one (1) patient (group B) used NuH Hair, 2% ketoconazole shampoo, and 1mg Propecia (finasteride) orally each day (Table 2). In one patient who used all four components (Group A), significant growth was achieved within 14 days (Figure 14). Three patients noted major growth at 30 days of treatment. Objective growth was noted in all eight patients by day no. 60 (Figures 1, 2, 3, 4, 5, 6, 7, and 8). 

**Figure 9:**
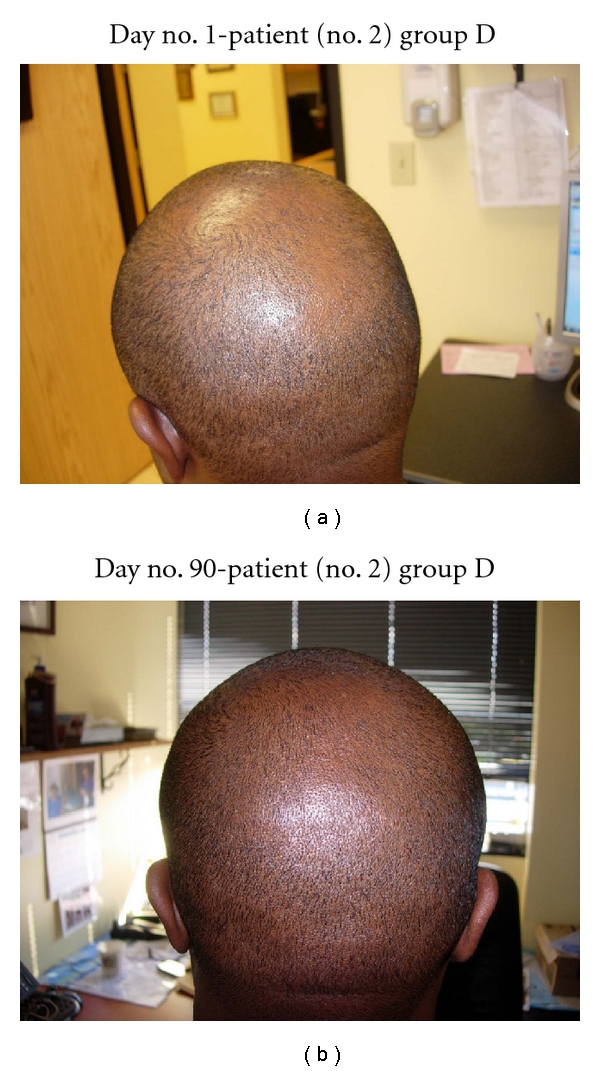
(a) Diffuse thinning of the vertex of the scalp. (b) At day no. 90, the vertex demonstrates much thicker hair and more uniform distribution of hair.

**Figure 10:**
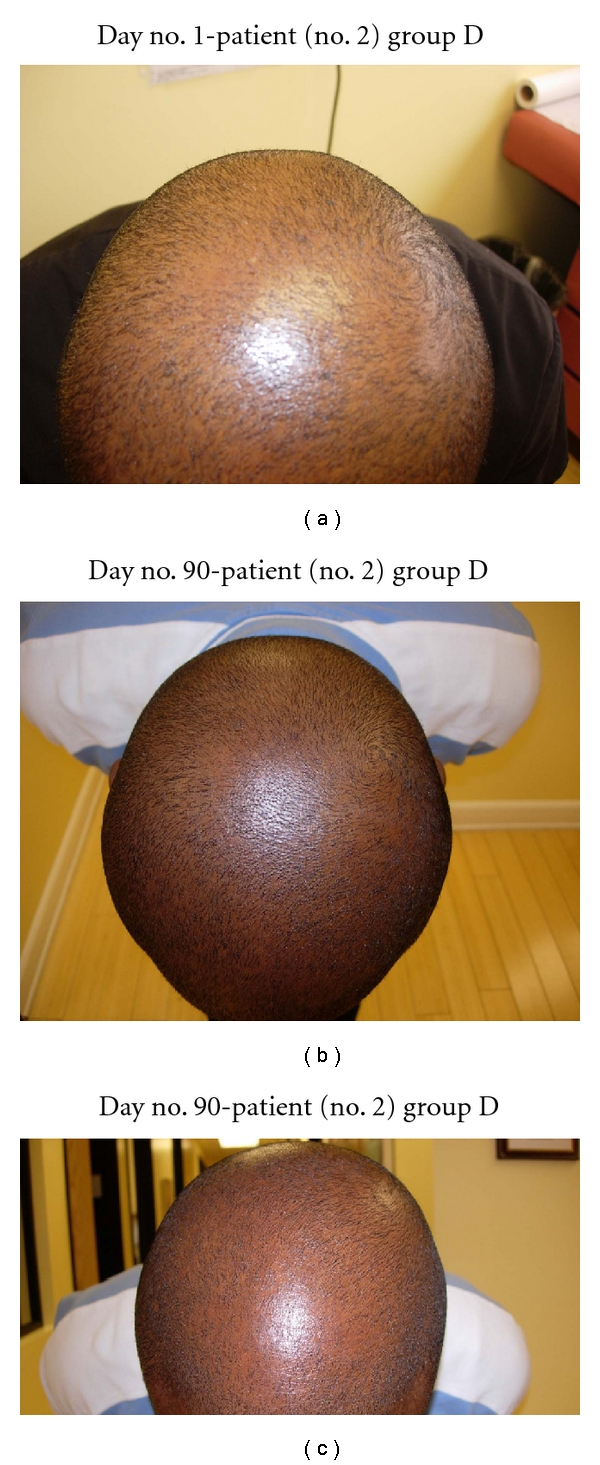
(a) Frontal scalp demonstrates thinning all the way back to the vertex. (b) At day no. 90, the frontal and vertex parts of the scalp are much more thick and uniform. (c) Another view with much less glare demonstrates a thick scalp vertex.

**Figure 11:**
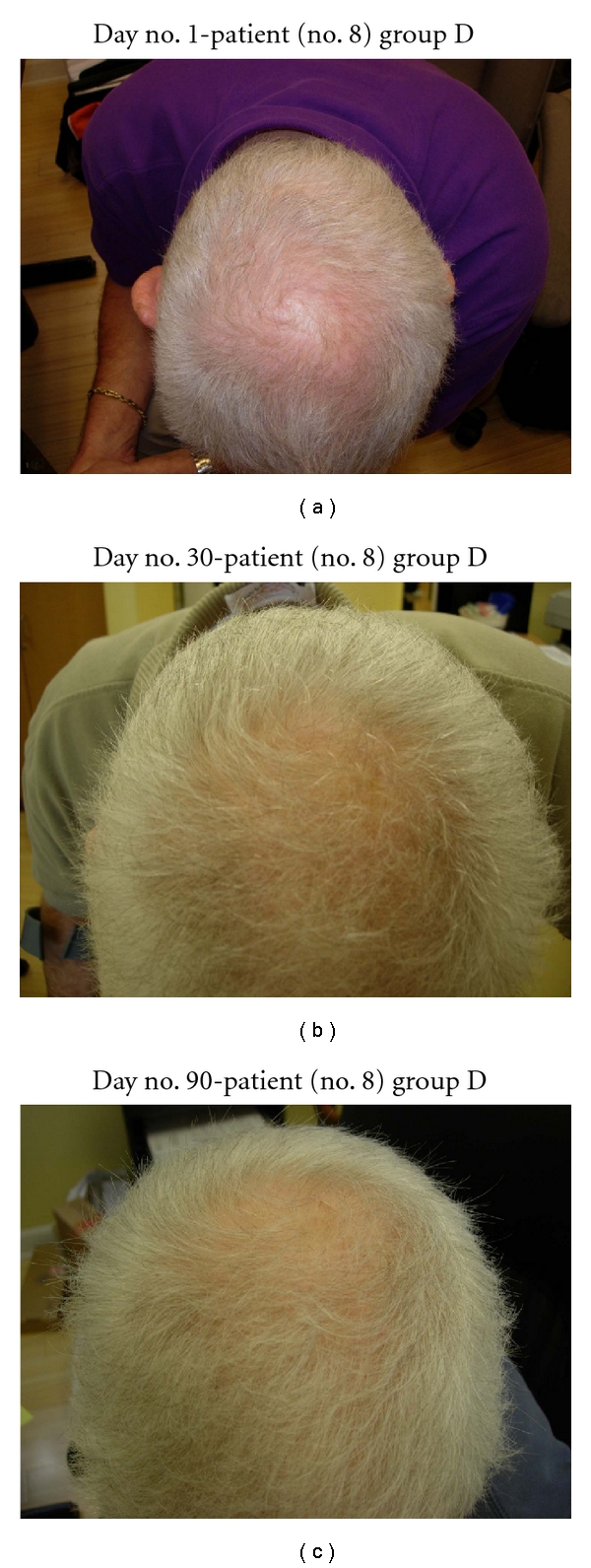
(a) Patient with significant thinning of the vertex. (b) At day no. 30, this patient demonstrates significant regrowth of scalp hair in the vertex area. This patient only used NuH Hair. (c) Another view of the scalp vertex at day no. 90.

**Figure 12:**
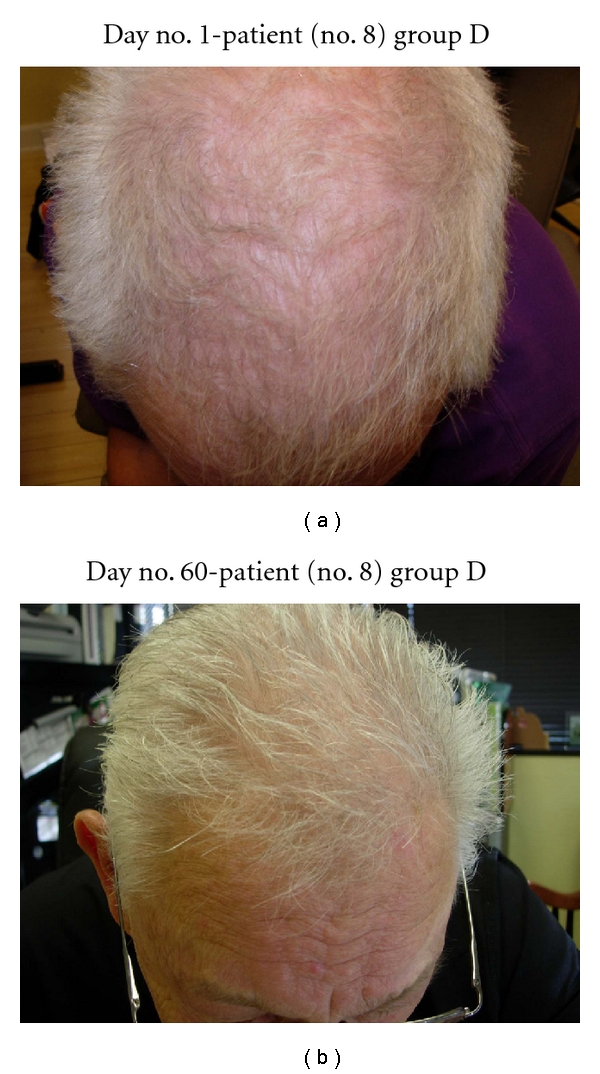
(a) Diffuse thinning of the frontal scalp noted at day no. 1. (b) At day no. 60, there is asignificant thickening of the frontal scalp hairs.

**Figure 13:**
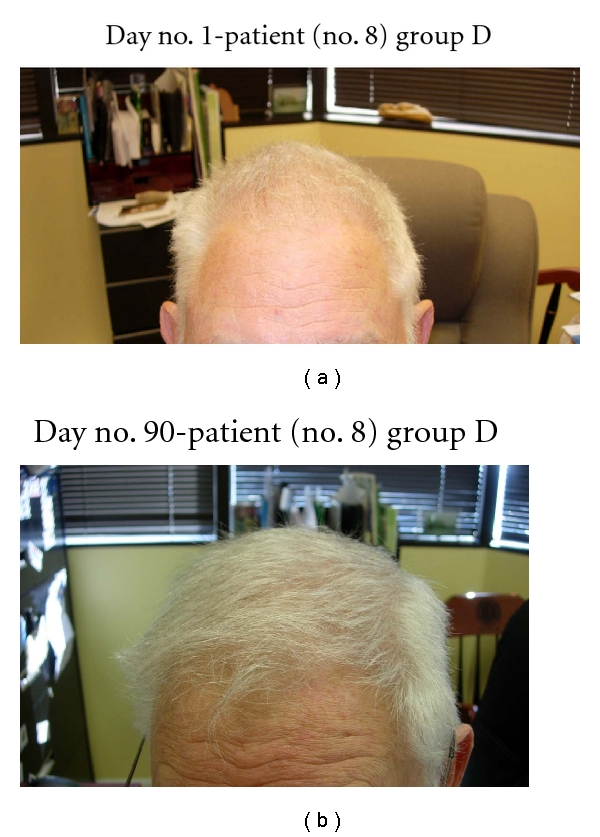
(a) At day no. 1, a thin recessed hairline is noted. (b) At day no. 90, the hairline is much thicker, and a full hairline is developing.

**Figure 14:**
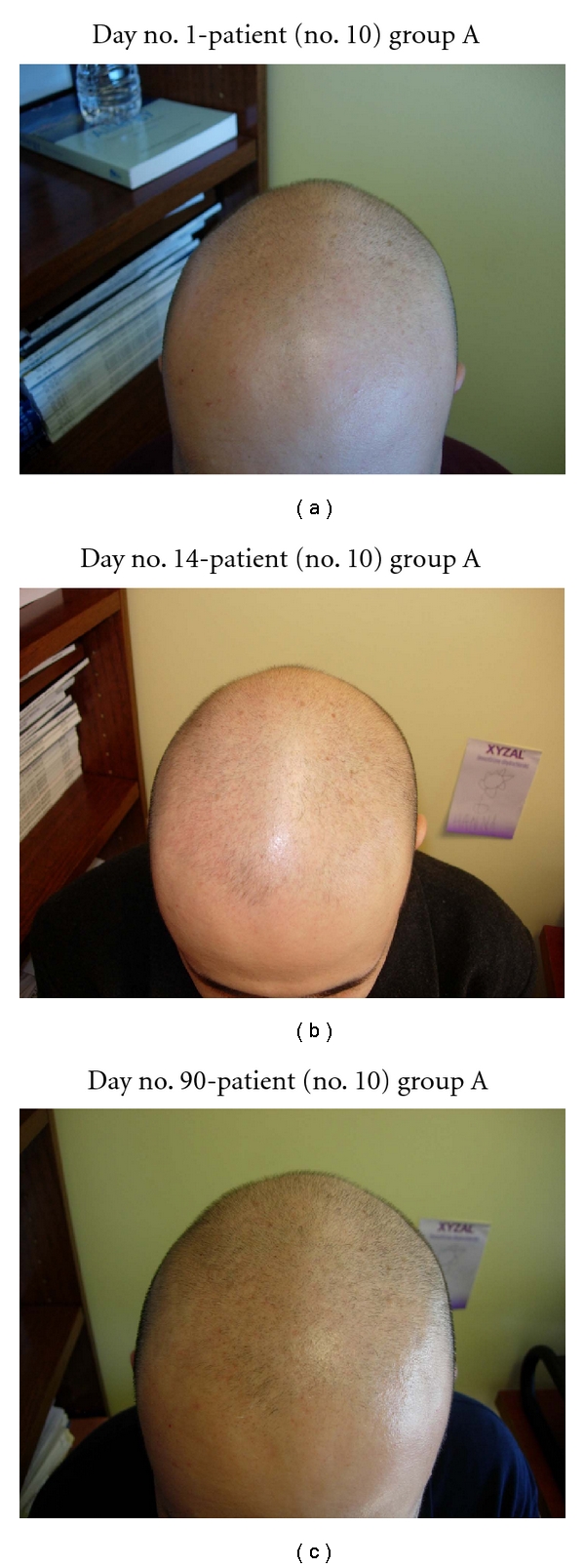
(a) Patient no. 10 with a 20-year history of AGA and 10-year history of stage 7 (Hamilton Norwood) AGA. (b) After only 14 days of the 4-part protocol, significant hair regrowth occurred at the right frontal scalp and hairline. (c) Diffuse hair growth noted at day no. 90 throughout the frontal scalp and vertex.

In the five patients who only used NuH Hair, all patients noted significant hair regrowth by 3 months of therapy (see Figures 9, 10, 11, 12, and 13). 

In this study, 10 patients incorporated 2% ketoconazole shampoo into their regimen. Eight of these patients had known seborrheic dermatitis (SD) and demonstrated significant improvement of their dermatitis after one month of therapy with 2% Nizoral shampoo. All of the patients with subjective complaints of scalp irritation, itching, and discomfort noted resolution of these symptoms within one month of 2% ketoconazole therapy. All patients were instructed to continue on the shampoo at least twice per week regardless of the resolution of their SD. In addition, all 10 patients who incorporated 2% ketoconazole shampoo expressed desire to continue treatment with the shampoo indefinitely.

One patient stopped our study after 60 days for logistic reasons. He demonstrated significant growth at day no. 55. This patient was only using NuH Hair and no other components (group D). He did not have any complaints of side effects with the study medication. His results were included as he demonstrated significant response.

Several patients in group D (NuH Hair alone) admitted to only using the Product 2–4 times per week. These patients still demonstrated significant growth despite not following the daily protocol (Figures 7, 8, 9, 10, and 11). 

## 4. Discussion

In this pilot study of 15 patients, all patients demonstrated new significant new hair growth after starting NuH Hair. The eight patients who implemented all 4 components of our protocol demonstrated the most significant and most rapid growth. In addition, one patient in group D noted significant regrowth 14 days after initiating therapy, and three of these patients noted hair growth as early as 30 days into treatment. This study, to our knowledge, is the first to demonstrate early significant hair regrowth in men with AGA. Our four-part protocol is also unique in that it addresses several underlying causes of hair loss in men simultaneously. This novel approach in treating hair loss is effective and represents an MPB treatment protocol that recommends use of four components simultaneously.

Those patients who utilized only NuH Hair also demonstrated significant regrowth of their hair. This was seen as early as 1 month into treatment in 2 patients, at 2 months in other 2 patients, and at 3 months in one patient. This patient group also contained subjects who admitted to only using the product 2-3 times per week during 3 months of therapy. This altered regimen was done on their own behalf, and not recommended by the investigators. It is important to note that because of dutasteride's 28-day half-life, less frequent application of topical dutasteride should be sufficient once steady-state concentration levels of dutasteride are present within the scalp. Because of the long half-life, this should theoretically be reached after 3–5 months of applying the solution on a daily basis. This is a secondary objective of our study and will be researched further over the following 12 months.

Patients who did not have signs or symptoms of seborrheic dermatitis also reported subjective improvement with use of 2% ketoconazole shampoo. Given ketoconazole's antiandrogen effects, it was expected to be effective in patients with and without seborrheic dermatitis.

With the use of 5% Rogaine and oral Propecia, a minimum of 4–6 months is required for significant clinical apparent hair growth. Most patients who use Rogaine and Propecia primarily experience a slowing of hair loss. Our patients not only experienced cessation of hair loss, but they also demonstrated significant hair growth beginning in 30 days, and certainly within 90 days. 

**Figure 15:**
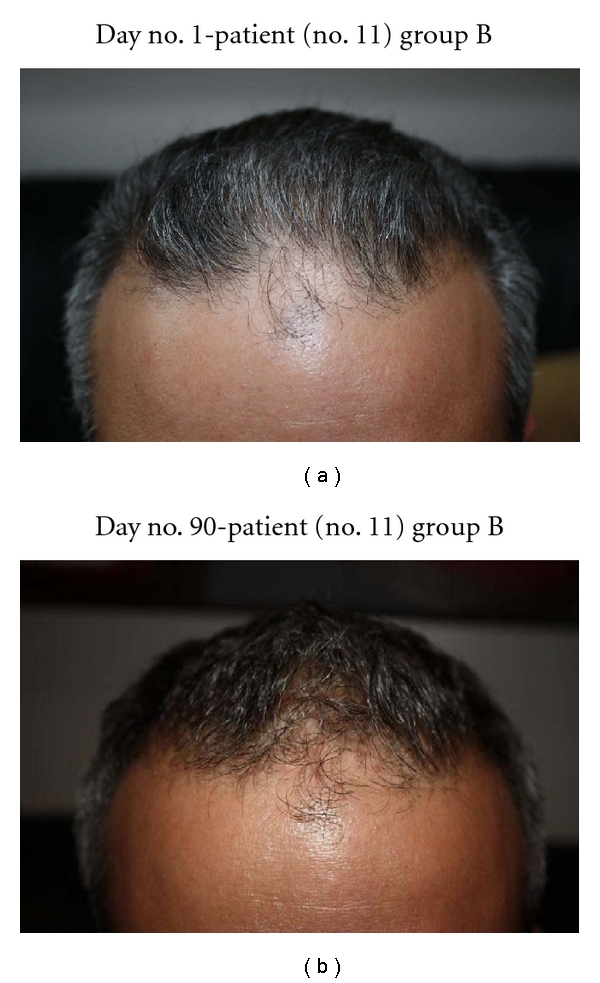
(a) Patient with diffuse irregular thinning of the anterior scalp and hairline. (b) Central frontal scalp hairline starting to fill in at 90 days.

**Figure 16:**
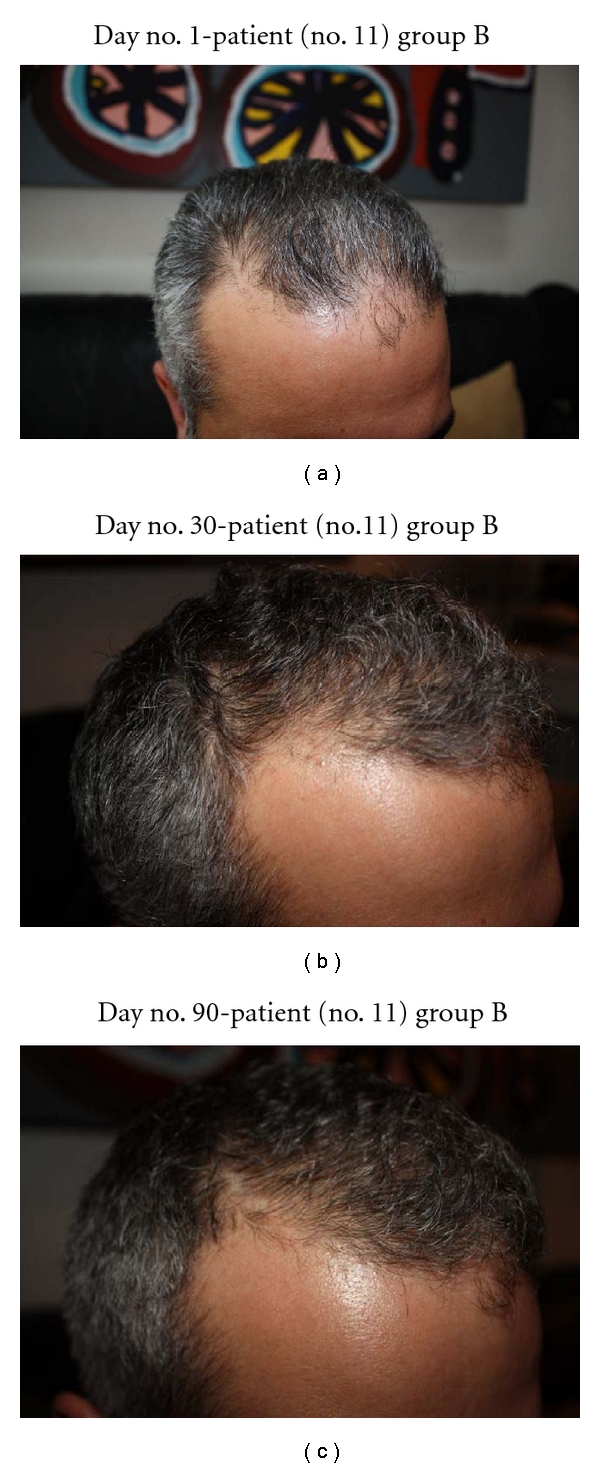
(a) Temporal scalp demonstrates recession. Another view of the anterior scalp and hairline. (b) After 30 days, this patient demonstrates regrowth at the temporal and anterior scalp. (c) Another view of the right temporal scalp and hairline, this time at day no. 90.

**Figure 17:**
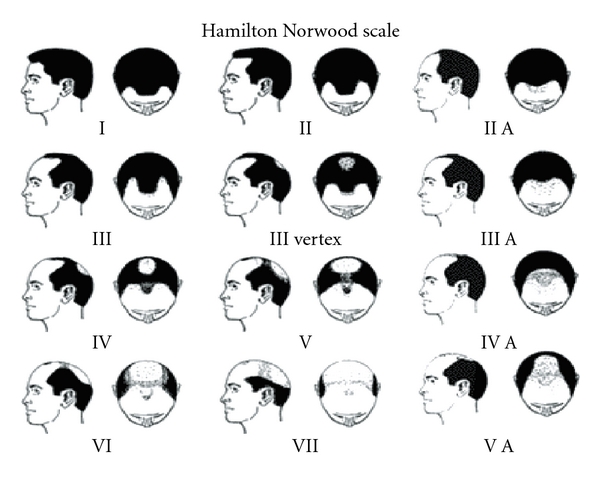


Interestingly, those patients who were smokers demonstrated the least significant hair regrowth. There was one patient in group A, and the other was in group B (Table 1; Figures 15-16). It is known that there is an association between men who smoke and premature AGA. The mechanisms of smoking-induced hair loss are multifactorial and include the effects of cigarette smoke on the dermal hair papilla microvasculature [21, 22]. 

All of the patients in group D (NuH Hair alone) were admitted to only use the product an average of 2-3 times per week. Despite this regimen, the patients still demonstrated significant hair regrowth. This was likely due to dutasteride's 28-hour half-life. This presents an interesting finding and may lead to further research with less frequent application of our topical product. 

There have been no side effects so far with the topical regimens noted in this study. The side effects of oral Propecia were a significant concern in many of our patients. Systemic side effects including erectile dysfunction, fatigue, breast enlargement, and decreased volume of semen were not noted in any of our study patients. All of our patients reported no systemic or local side effects.

## 5. Side Effects of Dutasteride

As with finasteride, similar side effects have been noted with dutasteride only when taken orally. These side effects include gynecomastia, decreased libido, ejaculation disorders, and impotence. These side effects were resolved in men who discontinued dutasteride and in most men who continued therapy. 

In addition, finasteride and dutasteride are absorbed through the skin. Therefore, women who are pregnant should not handle these capsules, as contact and inadvertent consumption could cause birth defects of the male fetus. Men should not donate blood while taking dutasteride. They should also wait 6 months, after treatment ends, before donating blood. The long period after discontinuing dutasteride is based upon a 28-day half-life. dutasteride can be carried in the blood and can cause birth defects if a pregnant woman receives a transfusion with blood that contains dutasteride. These are similar precautions for those patients taking finasteride. The absorption through the skin is significant, and this is why we chose to formulate a topical product to be applied to the scalp. 

Another concern in patients taking inhibitors of 5AR is the risk for prostate cancer. Earlier in this decade, the Prostrate Cancer Prevention Trial (PCPT) brought up concern for finasteride as a risk for higher-grade prostate cancer. In this study, despite a 25% reduction of prostate cancers in the treatment arm (those given 5 mg finasteride per day), significantly more high-grade cancers were demonstrated in this group. Further evaluation of this study demonstrated that this increased incidence was attributed to optimized tumor detection in smaller glands. Prostatic intraepithelial dysplasia (PIN) was also reduced in these patients [23, 24]. In another study, the REDUCE trial, a randomized controlled trial of 6,729 men compared dutasteride to placebo for the prevention of prostate cancer. These researchers found an overall reduction of 22% in the incidence of prostate cancer over 4 years. However, the reduction was entirely in Gleason grades 5 and 6 cancers, which are less lifethreatening and often not treated. In Gleason grades 7–10, which are lifethreatening, there was no reduction in cancer [23, 24]. 

## 6. Conclusion

Our study was a prospective pilot study in 15 patients. It presents a novel technique at treating MPB with an aggressive multifaceted approach. The study is limited in that we did not conduct a double-blind, placebo-controlled study. However, our study was conducted as a classic pilot study. Our goal was to design a preliminary study prior to our main research, in order to check the feasibility and to improve the design of our research. This goal was met, and it should serve as a foundation to further investigate this product in a large sample, double-blind, placebo-controlled prospective study for MPB. 

## Figures and Tables

**Table 1:** Patient demographics.

Initials	Study group	Age	Atopic	Concomitant disorders	Smoker	Hamilton Norwood Scale
KC	D	33	No	NONE	NO	IV
JW	D	38	No	NONE	NO	IV/V
RR	A	24	Yes	SD	NO	VII
AR	A	38	Yes	AD, SD	NO	II
SS	D	50	No	NONE	NO	III
HC	C	38	Yes	SD, AD**	NO	V
CK	A	43	Yes	SD	NO	VII
RK	D	72	No	MS*	NO	III
NC	A	42	Yes	SD, AD**	NO	VII
JG	A	32	Yes	SD	NO	VII
AR	B	38	No	NONE	YES	III
AH	D	45	Yes	SD	NO	VII
RA	A	30	Yes	SD	YES	V
WM	A	59	Yes	SD	NO	II
JH	A	36	Yes	SD	NO	IV

*Taking copaxone which is known to cause alopecia.

SD: Seborrheic dermatitis.

AD: Atopic dermatitis.

MS: Multiple sclerosis.

**Severe atopic dermatitis.

**Table 2:** Patient subgroups.

	Group A	Group B	Group C	Group D
Components	NuH Hair, Propecia 1 mg/day, Rogaine Foam each AM, 2% ketoconazole shampoo BIW/TIW	NuH Hair, 2% ketoconazole shampoo BIW/TIW, Propecia 1 mg/day	NuH Hair, 2% ketoconazole shampoo BIW/TIW	Nuh Hair
No. of patients	8	1	1	5
Presence of seborrheic dermatitis	8	1	1	0
Average time for hair growth (days)	30 days	30 days	60 days	90 days
